# Case report: nosocomial fungemia caused by *Candida diddensiae*

**DOI:** 10.1186/s12879-020-05095-3

**Published:** 2020-05-27

**Authors:** Seong Eun Kim, Sook In Jung, Kyung-Hwa Park, Yong Jun Choi, Eun Jeong Won, Jong Hee Shin

**Affiliations:** 1 Department of Infectious Diseases, Chonnam National University Medical School, 42, Jebong Ro, Donggu, Gwangju, 61469 Republic of Korea; 2 Department of Laboratory Medicine, Chonnam National University Medical School, Gwangju, Republic of Korea

**Keywords:** *Candida diddensiae*, Fungemia, Leukopenia, Malignancy, Total parenteral nutrition

## Abstract

**Background:**

*Candida diddensiae*, a yeast found in olive oil, is considered non-pathogenic to humans. Here, we describe the first case of fungemia caused by *C*. *diddensiae* in a hospitalized patient with underlying diseases.

**Case presentation:**

A 62-year-old woman was admitted because of multiple contusions due to repeated falls and generalized weakness. She presented with chronic leukopenia due to systemic lupus erythematosus, and multiple cranial nerve neuropathies due to a recurring chordoma. She was given a lipid emulsion containing total parenteral nutrition (TPN) starting on the day of admission. Broad-spectrum antibiotics had been administered during her last hospital stay and from day 8 of this hospitalization. However, no central venous catheter was used during this hospital stay. Blood cultures obtained on hospital days 17, 23, and 24 yielded the same yeast, which was identified as *C*. *diddensiae* via sequence analyses of the internal transcribed spacer region and D1/D2 regions of the 26S ribosomal DNA of the rRNA gene. In vitro susceptibility testing showed that the minimum inhibitory concentration of fluconazole for all isolates was 8 μg/mL. On day 23, TPN was discontinued and fluconazole therapy was started. Blood cultures obtained on day 26 were negative. The fluconazole therapy was replaced with micafungin on day 26 and the patient exhibited improvements.

**Conclusion:**

The use of lipid TPN may potentially contribute to the occurrence of nosocomial fungemia by *C. diddensiae*, an unusual *Candida* species.

## Background

Due to an increasing number of patients with impaired immune function and developments in medical interventions, some *Candida* species previously considered to be food yeasts or harmless commensals are emerging as causes of invasive disease [1]. *Candida diddensiae* has been considered non-pathogenic to humans but is a component of the olive oil microbiota that affects the quality of the oil [2]. Although rare, this organism has been isolated from the human gut [3, 4], and one clinical isolate of *C*. *diddensiae* is included in a recent national surveillance dataset of invasive candidiasis in China [5]. However, no detailed clinical data have been presented, thus the potential of this organism for human infection remains unclear. Here, we describe a case of *C*. *diddensiae* fungemia in a hospitalized patient with intractable cancer and chronic leukopenia. To our knowledge, this is the first reported case of *C*. *diddensiae* fungemia.

## Case presentation

**Fig. 1:**
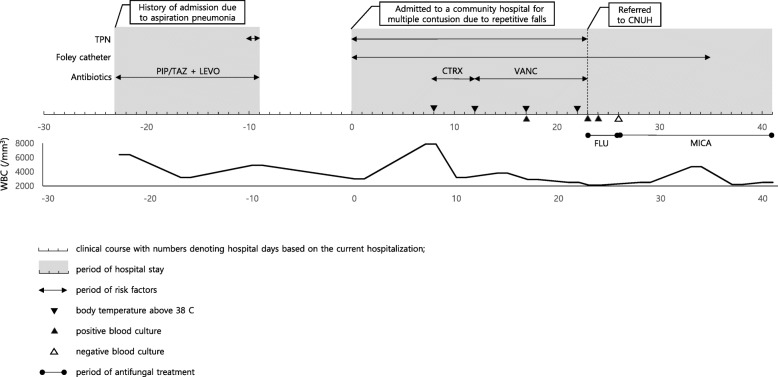
Clinical course and treatment of the patient with *C*. *diddensiae* fungemia. TPN, total parenteral nutrition; PIP/TAZ, pipera cillin/tazobactam; LEVO, levofloxacin; CTRX, ceftriaxone; FLU, fluconazole; MICA, micafungin; WBC, white blood cell; CNUH, Chonnam National University Hospital

A 62-year-old woman was admitted to a community hospital in Jangheung, South Korea for multiple contusions due to repeated falls. After admission, she was treated with antibiotics, urinary catheterization, and a commercial lipid emulsion-containing total parenteral nutrition (TPN). *C*. *diddensiae* fungemia was diagnosed based on positive blood cultures drawn from peripheral veins on hospitalization days 17, 23, and 24 (Fig. 1).

The patient had an 8-year history of systemic lupus erythematosus (SLE). From the time of SLE diagnosis, she had been taking methylprednisolone, azathioprine, and hydroxychloroquine sulfate and tapered them gradually. Her SLE has been well controlled without any medication for the past 2 years. Despite good control of SLE, chronic leukopenia of 2000 to 3000/mm^3^ (neutrophils 600 to 1500/mm^3^) persisted, and bone-marrow biopsy revealed hypocellularity of all cell lines without dyserythropoiesis or megakaryocytic atypia, which is typical of SLE. The patient had been diagnosed with a chordoma in the sphenoid sinus and clivus 5 years earlier. Despite repeated neurosurgery, the tumor progressed and resulted in multiple cranial nerve neuropathies. One month before hospitalization, the patient reported worsened difficulty in swallowing, and a physical examination revealed right vocal cord palsy and no right oropharyngeal lateral wall movement. Twenty-three days before this admission, she was admitted for aspiration pneumonia and given piperacillin/tazobactam and levofloxacin for 2 weeks. She has never received antifungal treatment.

A lipid emulsion containing TPN was started for nutrition support on day 1 of hospitalization and all infusions were completed within 24 h of initiating the fluid. On day 8, the patient developed an intermittent fever (peak temperature 38.8 °C) that persisted for 14 days. Catheter-associated urinary tract infection was suspected and ceftriaxone was initiated after a urine culture was taken. The antibiotic was subsequently changed to vancomycin because the urine culture revealed the presence of ampicillin-resistant, vancomycin-susceptible *Enterococcus faecium*. Chest and abdominal computed tomography did not reveal any focus of infection or perforation. Because blood cultures obtained on day 17 indicated the presence of a yeast, she was transferred to Chonnam National University Hospital (CNUH; Gwangju, South Korea) for the treatment of fungemia. Upon physical examination, there were no local inflammatory signs at the peripheral intravenous catheter sites, such as erythema, swelling or tenderness. On arrival at CNUH (on day 23), a follow-up blood culture was performed, and intravenous fluconazole was initiated empirically (800 mg loading and then 400 mg/day). The TPN was discontinued. An ophthalmological examination showed no evidence of endophthalmitis, and transthoracic echocardiography showed no evidence of endocarditis. On day 26 (4 days after CNUH admission), the fluconazole was replaced with micafungin (100 mg/day for 16 days) after 4 days of fluconazole treatment because of the high fluconazole minimum inhibitory concentration (MIC) in antifungal susceptibility tests. Follow-up blood cultures taken on day 26 were negative. The patient’s condition improved and she was discharged on day 41 after supportive care and intravenous echinocandin treatment.

Six blood culture sets from peripheral blood obtained on days 17, 23, and 24 (two sets on each day) yielded the same yeast isolate. The isolate grew on sheep blood agar as non-hemolytic, white colonies after 48 h of incubation at 35 °C in an aerobic environment, and yielded pink colonies on BBL CHROMagar Candida (BD Diagnostics, Franklin Lakes, NJ, USA). The isolates were analyzed by sequencing the internal transcribed spacer (ITS) and D1/D2 regions of the 26S ribosomal DNA of their rRNA gene. The ITS region (including the 5.8S rRNA gene) and the D1/D2 domains of the 26S rRNA gene were amplified using the primer pairs pITS-F (5′-GTCGTAACAAGGTTAACCTGCGG-3′) and pITS-R (5′-TCCTCCGCTTATTGATATGC-3′) and NL1 (5′-GCATATCAATAAGCGGAGGAAAAG-3′) and NL4 (5′-GGTCCGTGTTTCAAGACGG-3′) [6], respectively. The yeast was identified as *C*. *diddensiae* (GenBank accession no: KC253978 and MK394116.1) with 100% homology using BLAST (www.ncbi.nlm.nih.gov/blast). The isolate was misidentified as *Millerozyma farinosa* (93% probability) when the Vitek2 YST card (bioMérieux, Marcy l’Étoile, France) systems was used. Two commercial matrix-assisted laser desorption/ionization-time of flight mass spectrometry (MALDI-TOF MS) systems, the VITEK MS (bioMérieux) with Knowledge Base ver. 3.0 and the MALDI-TOF Biotyper (Bruker Daltonics, Billerica, MA, USA) were not able to identify the yeast because *C*. *diddensiae* was not included in their databases. Antifungal susceptibility testing performed using the broth microdilution M27-A3 method of the Clinical and Laboratory Standards Institute (CLSI) produced following MICs: amphotericin B, 1 μg/mL; fluconazole, 8 μg/mL; voriconazole, 0.25 μg/mL; caspofungin, 0.25 μg/mL; and micafungin, 0.125 μg/mL.

## Discussion and conclusion

*Candida* species are a members of human mucosal and skin microbiotas, and most cases of fungemia involve endogenous flora [7]. However, *C*. *diddensiae* is not commonly found in healthy individuals. *C*. *diddensiae* was reported for the first time in extra virgin olive oil [2]. Recent research has demonstrated the presence of a rich microflora (mainly yeasts) in freshly produced olive oil [8]. In addition, *C*. *diddensiae* is a resident eukaryote in the infant gastrointestinal tract [3, 4, 9]. Our patient did not have a central venous catheter, so this food-associated fungus most likely entered through the intestinal tract with the consumption of contaminated food. Although *C*. *diddensiae* colonization of the gastrointestinal tract before candidemia was not documented in this patient, broad-spectrum antibiotics might have affected the gut microbiota and the level of yeast colonization of the gastrointestinal tract [10, 11]. The TPN infusion, broad-spectrum antibiotic use, and long-term neutropenia caused by SLE might have led to nosocomial fungemia by *C*. *diddensiae* in our patient with a recurring progressive chordoma.

Because the TPN infusate and peripheral catheters were not cultured, contamination of the TPN set or a peripheral catheter infection could not be excluded. However, the TPN sets were all changed within 24 h of initiating the fluid and the peripheral catheters were replaced within 96 h based on the guidelines for the prevention of intravascular catheter-related infection [12]. There were also no local signs of inflammation at the peripheral intravenous catheter sites, such as erythema, swelling, or tenderness. The TPN formula used for this patient contained a lipid emulsion. Although the role of lipid addition to TPN in fungal infections is not well-elucidated [13], recent research has shown that lipid emulsions induce *Candida* virulence determinants, such as germination and enhanced biofilm production, which may help to explain the increased risk of candidemia [14]. Our patient produced persistent positive blood cultures for *C*. *diddensiae* while she was receiving lipid TPN. Although the blood isolates had a relatively high fluconazole MIC (8 μg/mL), the candidemia cleared after 3 days of fluconazole and the discontinuation of TPN. Furthermore, the soybean oil of the lipid emulsion containing TPN and olive oil are composed of similar components [15, 16]. These findings suggest that the lipid emulsion containing TPN might increase the risk of *C*. *diddensiae* fungemia by providing a favorable lipid-rich environment for *C*. *diddensiae* survival in the blood. Therefore, this case raises concerns about the potential contribution of lipid-emulsion-containing TPN use to the occurrence of *C*. *diddensiae* bloodstream infections.

The incidence of invasive fungal disease in patients with SLE is 0.6–3.2% [17, 18]. Invasive fungal disease is more likely to develop during the active stage of SLE. Although this patient was not at the active stage of SLE, long-lasting leukopenia caused by SLE induced bone-marrow failure and the resulting immune impairment might have contributed to the dissemination of candidemia [19–21]. Furthermore, leukopenia is a risk factor for candidemia caused by non-albicans *Candida* species compared with *C. albicans* [22, 23]. These findings highlight the pathogenic potential of a relatively avirulent organism in the setting of immunosuppression.

Mycology laboratories are challenged by the identification and antifungal susceptibility testing of unusual, clinically significant yeasts. Our case shows that *C*. *diddensiae* cannot be correctly identified with commercial identification systems or two MALDI-TOF systems used in routine microbiology laboratories, because of the absence of this organism from their databases. Molecular methods were required for accurate identification.

The use of azole as a fungicide in agriculture and the persistence of fungicides in the environment promote the emergence and spread of azole-resistant fungi [24]. Several common *Candida* species that cause human disease, such as *C. tropicalis*, *C. famata*, *C. guilliermondii*, *C. krusei*, *C*. *orthopsilosis*, and *C. parapsilosis*, were detected on the surface of fruit purchased from supermarkets and 39% had a fluconazole MIC of ≥8 mg/L [25]. Although the CLSI has not established species-specific clinical breakpoints for *C*. *diddensiae*, the *C*. *diddensiae* isolated from the present case had a fluconazole MIC of 8 mg/L, consistent with a previous report [5]. Therefore, uncommon *Candida* species isolated from blood samples should be identified and antifungal susceptibility testing performed to select the appropriate antifungal therapy.

This case suggests that *C*. *diddensiae*, which is considered non-pathogenic to humans and of industrial importance only, can be a pathogen and has the potential to cause bloodstream infection in immunocompromised patient. Unusual *Candida* species may vary greatly in their susceptibility to current antifungal agents, which can cause considerable issues in patient management [26].

## Data Availability

Not applicable (no datasets were generated or analyzed during this study).

## Abbreviations

TPN: total parenteral nutrition
SLE: systemic lupus erythematosus
MALDI-TOF MS: matrix-assisted laser desorption/ionization-time of flight mass spectrometry
CLSI: Clinical and Laboratory Standards Institute
MIC: minimum inhibitory concentration
CNUH: Chonnam National University Hospital
